# Loop-Mediated Isothermal Amplification (LAMP) as a Rapid, Affordable and Effective Tool to Involve Students in Undergraduate Research

**DOI:** 10.3389/fmicb.2020.603381

**Published:** 2020-12-09

**Authors:** Andrew V. Nguyen, Amos Orlofsky, Kaylynn Pubill, Mangala Tawde, Gaozhen Li, Diana Mata, Oscar Bermudes, Miguel Fernandez, Jonathan Santana, Woochul Kim, Enzon Chimbay, Yeeun Kim, Trieu Nguyen, Malcolm Fox, Janelly Eralte, Molly Metz, Davida S. Smyth, Caterina Panzeca, Mazhar I. Khan

**Affiliations:** ^1^Department of Biological Sciences and Geology, The City University of New York, Queensborough Community College, Bayside, NY, United States; ^2^Department of Natural Sciences, Mercy College, Dobbs Ferry, NY, United States; ^3^Department of Natural Sciences and Mathematics, Eugene Lang College of Liberal Arts of the New School, New York, NY, United States; ^4^Department of Science, SUNY Maritime College, New York, NY, United States; ^5^Department of Pathobiology and Veterinary Science, College of Agriculture, Health and Natural Resources, Storrs, CT, United States

**Keywords:** LAMP, research tool, undergraduate research (UR), HIP’s, stem education

## Abstract

Undergraduate research (UR) is a high-impact practice (HIP) to engage undergraduate student in science, technology, engineering and mathematics (STEM), especially from underrepresented groups. UR experiences (UREs) can be integrated into the classroom, making authentic research experiences inclusive and available to all students. However, developing UR pedagogy can be challenging for faculty in resource-limited labs, such as community colleges and small liberal arts colleges. Often molecular biology research methods are expensive, time-consuming and need equipment not readily available or affordable in small schools. Polymerase chain reaction (PCR) is one of the most commonly used techniques in research labs and many UREs. We have investigated loop-mediated isothermal amplification (LAMP) as an inexpensive, accessible alternative to PCR for DNA amplification enabling the identification of microorganisms in the context of UREs. LAMP does not require expensive instrumentation or reagents and uses equipment commonly found in teaching labs. By performing the technique, students learn several key scientific skills that will be useful in their undergraduate or graduate STEM careers. We designed guided independent research experiences for several undergraduates that included the use of LAMP. Students successfully applied the technique to culture samples of common environmental bacteria, including *Escherichia coli*, *Salmonella* spp., *Staphylococcus aureus*, and *Enterococcus*, and were in addition, able to detect both *Salmonella* and *Enterococcus* in directly sampled environmental waters. To highlight the accessibility and affordability of this URE, a simple boiling method was used for DNA preparation from environmental samples. Student response data show positive attitudes toward UR when LAMP is utilized as a research tool to tackle relevant biological questions. The feasibility of using simplified LAMP in UREs points to a potential, more expanded application to public engagement with science and broader and more inclusive interactions with the research community.

## Introduction

The Association of American Colleges and Universities has recognized several instructional modalities as high-impact practices (HIPs) that increase student retention rates and student engagement (Kuh, 2008). Among these practices, undergraduate research (UR) has been found to be a particularly effective pedagogy for the engagement and retention of undergraduate students (Kuh, 2008). UR can provide students with not only scientific skills but also increased self-confidence, improved oral and written presentation skills and enhanced critical thinking (Kuh and O’Donnell, 2013).

Two main challenges are faced when creating UR experiences (UREs). First, it may be difficult to design projects that are realistically feasible and that also engage students in relevant authentic queries. Second, many procedures require reagents, equipment and/or central technical support that are beyond the means of many undergraduate institutions. One category of studies that can address the first issue consists of projects that focus on environmental sampling, such as the identification of pathogens from environmental samples like soil and water. The current COVID-19 pandemic has heightened student awareness of the value of such studies. However, environmental pathogen identification typically involves the use of polymerase chain reaction (PCR) for DNA amplification, which is time consuming, expensive and can be difficult for students to perform accurately. At Queensborough Community College (QCC) we have developed UREs in which loop-mediated isothermal amplification (LAMP) can be used as a simpler, sensitive and far less expensive alternative to PCR for the identification of microbes in samples obtained from locations on campus as well as from the surrounding urban environment. While comparable in its value as a teaching tool to PCR, LAMP is over tenfold less expensive, as well as more sensitive and more robust in handling complex biological samples (Kaneko et al., 2007; Mori and Notomi, 2009; Law et al., 2014; Warghane et al., 2017). The simplicity of colorimetric readouts, coupled with the simplicity of the amplification procedure itself, makes it easy to incorporate LAMP in a classroom context, so that the method can be used to support course-based UREs (CUREs) as well as independent UR projects.

The standard LAMP assay does not require equipment beyond those that are available in standard biology laboratories (see Supplementary Figure S1). The simplicity of the setup derives from the dependence of LAMP on the inherent strand-displacing activity of the Bst polymerase, avoiding the need for the repeated high-temperature intervals employed in PCR, so that the entire reaction can be performed at a single temperature (60–68°C), using a non-denatured double-stranded DNA template (see Supplementary Figure S2). However, sample preparation methods prior to the assay may still consume classroom time and require specialized equipment and/or expensive supplies. By using the simple boiling method of sample preparation, LAMP can be made accessible not only to on-campus undergraduates but also to the at-home student in the context of distance learning, now emerging as a dominant mode of instruction during the COVID-19 pandemic. Even beyond the at-home student, we suggest that simplified LAMP protocols that avoid or minimize sample preparation may be uniquely positioned to promote a broader scientific engagement with lay communities and foster enhanced interactions between the research community and the public.

We describe here some initial illustrative data generated by several of our undergraduates with whom we designed guided UREs. These students were able to use LAMP to detect several bacterial microorganisms commonly found in the environment: *Escherichia coli (E. coli)*, *Salmonella* spp. (*S.* spp.), *Staphylococcus aureus (S. aureus)*, methicillin-resistant *Staphylococcus aureus* (MRSA), and *Enterococcus* (Hill et al., 2008; Tang et al., 2012; Wang et al., 2015; Lin et al., 2017; Hu et al., 2018).

## Materials and Methods

### Setting the URE

The students who participated part in the URE were either Biotechnology or Chemistry majors in an urban community college who had enrolled in a research laboratory internship course. Students had taken two semesters of introductory biology as well as biotechnology either prior to or concurrent with the URE.

### Bacterial Strains and Growth Media

*Escherichia coli*, *Salmonella* spp., *Staphylococcus aureus*, *Enterococcus faecalis*, and methicillin-resistant *Staphylococcus aureus* (MRSA) were grown in Brain Heart Infusion Broth or Tryptic Soy Broth.

### Sample Preparation

Soil samples (approximately 5 ml) were suspended in 50 ml water in conical tubes and allowed to settle prior to removal of 10 ml supernatant for DNA extraction by the boiling method (De Medici et al., 2003). DNA extraction from standing water samples (10 ml) was carried out by the boiling method. For *Enterococcus* tests, samples (10 ml) were collected from the East River (40.80557°N, 73.79661°W) and extracted by the boiling method.

DNA preparation was either by column-based purification (Quick-DNA Microprep Kit, Zymo Research, Irvine, CA, United States) or by the boiling method (De Medici et al., 2003). For the boiling method, all samples were first spun and resuspended at 1/10^*th*^ original volume.

### LAMP Assay

The target genes and protocols employed in the study are summarized in Table 1. Note that *fimY* (encodes for the Fimbriae Y protein) and *nucA* (encodes for the nuclease) are conserved targets for all serotypes of *Salmonella* and *S. aureus*, respectively, while *safA* (encodes for the major subunit of *S. enterica* atypical fimbriae) is specific for *Salmonella enteritidis*. *malB* (encodes for maltose operon protein B) is specific to *E. coli* and *femA* (encodes for protein that affect the level of methicillin resistance) is specific for methicillin-resistant *S. aureus* (MRSA).

**TABLE 1:** Target genes and protocols for LAMP assays.

	**Target gene**	**Incubation temperature**	**References**
*E. coli*	*malB*	66°C	Hill et al. (2008)
*Salmonella* spp.	*fimY*	65°C	Tang et al. (2012)
*Salmonella enteritidis*	*safA*	65°C	Azinheiro et al. (2018)
*S. aureus*	*nucA femA* (MRSA)	60°C 60°C	Wang et al. (2015) Lin et al. (2017) Chen et al. (2017)
*Enterococcus* spp.	23S rRNA	64°C	Martzy et al. (2017)

All LAMP incubations were for 1 hour. Reaction products were visualized by ethidium bromide staining of agarose gels. The amplified products from the LAMP reaction are not single-size amplicons but rather exhibit a range of different product sizes (Supplementary Figure S2, Part 5b). Thus, a positive LAMP reaction appears as a smear or a ladder of amplified products on an agarose gel, rather than a single band as seen with PCR (Hill et al., 2008; Tang et al., 2012; Wang et al., 2015; Lin et al., 2017; Hu et al., 2018). For detection of *Enterococcus* spp. in environmental samples, the LAMP amplification of the 23S rRNA target was performed with the addition of SYBR Green I, and the reaction was performed for 60 min in a qPCR machine (Quantstudio 6, Applied Biosystems in order to quantitate fluorescence each minute as a measure of product yield).

### Detection of *Enterococcus* by Enterolert

The Enterolert test (IDEXX Laboratories, Westbrook, Maine) is an enzymatic reaction based on the ability of *Enterococcus* beta-glucosidase to cleave 4-methyl-umbelliferyl b-D-glucoside, yielding a fluorescent product. Briefly, water samples for enumeration of *Enteroccoci* via IDEXX Enterolert Media were collected from the East River (40.80557°N, 73.79661°W) in 1 L acid washed high-density polyethylene bottles. Samples were processed the same day per the manufacturer’s protocol. After incubation at 41°C for 24 h, samples were read using a 6-watt, 365 nm UV light box, and enumerated using the IDEXX MPN table.

### Laboratory Safety

The URE in which students participated includes education in the biology and clinical impact of the pathogens studied. Standard biosafety practices for microbiology laboratory work were enforced, including adherence to Biosafety Level 2 (BSL2) procedures. Aseptic technique with a Bunsen burner was used for bacterial work. No enrichments of environmental samples were performed, although students were made aware that these samples can contain disease-causing organisms.

## Results

### Amplification From DNA of Known Strains

#### Optimization

**FIGURE 1:**
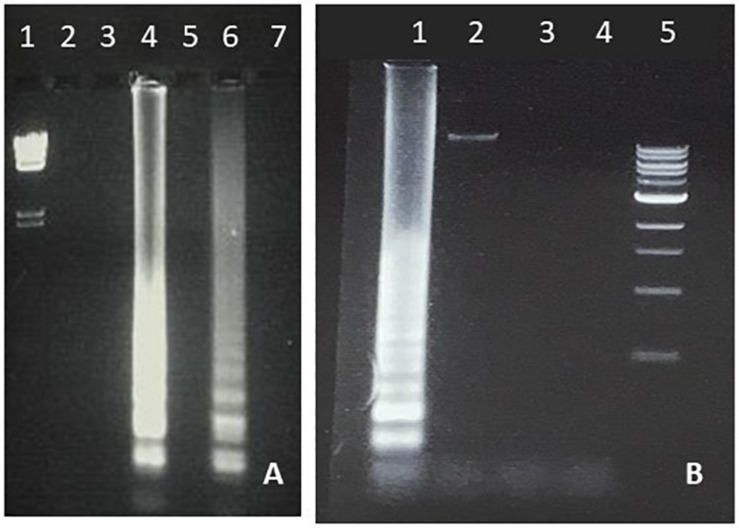
LAMP amplification of *E. coli.* **(A)** Temperature optimization of *E. coli* LAMP. Lane 1, lambda *Hin*dIII marker. Lanes 2, 4, and 6 were generated with chromosomal DNA and Lanes 3, 5, and 7 with water as negative control. Lanes 2 and 3 were incubated at 70°C, Lanes 4 and 5 at 66°C and Lanes 6 and 7 at 62°C. **(B)** Specificity of *E. coli* LAMP. Lane 1; *E. coli*, Lane 2; *S. typhimurium*, Lane 3; *Staphylococcus aureus*, Lane 4; water, Lane 5; 1 Kb marker. All reactions were carried out at 66°C.

**FIGURE 2:**
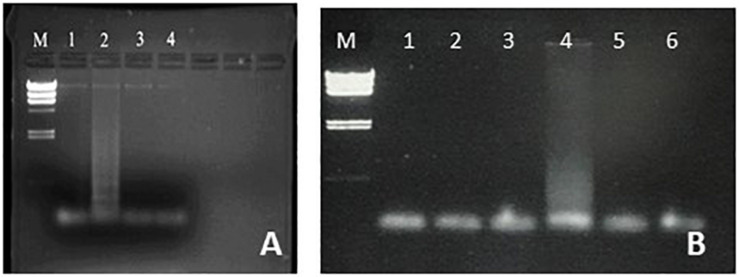
Optimization of *S. aureus* LAMP amplification. **(A)** Optimization of LAMP targeting the *femA* gene. Lane M, lambda *Hin*dIII marker. Reactions carried out at 55°C (lane 1), 60°C (lane 2), 65°C (lane 3), and 70°C (lane 4). **(B)** Optimization of LAMP targeting the *nuc* gene. Lane M, lambda *Hin*dIII marker. Lanes 2, 4, and 6 have *S. aureus* template and lanes 1, 3 and 5 are negative controls with water in place of template. Lanes 1, 2: 57°C. Lanes 3, 4: 62°C. Lanes 5, 6: 67°C.

**FIGURE 3:**
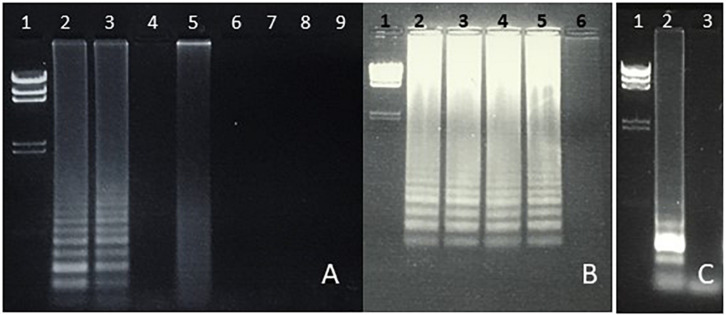
(**A, B)** Specificity of LAMP amplification of *Salmonella* spp. (*fimY* target). **(A)** Lane 1, lambda *Hin*dIII marker. Lane 2, *S. typhimurium*. Lane 3, *S. Newport.* Lane 4, *E. coli.* Lane 5. *Enterococcus* (*Streptococcus) faecalis.* Lane 6, *S. aureus.* Lane 7, *Proteus vulgaris*. Lane 8, *Serratia marcescens*. Lane 9, water (negative control). **(B)** Lane 1, lambda *Hin*dIII marker. Lane 2, *S. typhimurium*. Lane 3, *S. heidelberg*. Lane 4, *S. enteritidis*. Lane 5, *S. Newport*. Lane 6, water (negative control). **(C)** LAMP amplification of *S. enteritidis* (*safA* target). Lane 1, Lambda *Hin*dIII marker. Lane 2, *S. enteritidis*. Lane 3, water.

**FIGURE 4:**
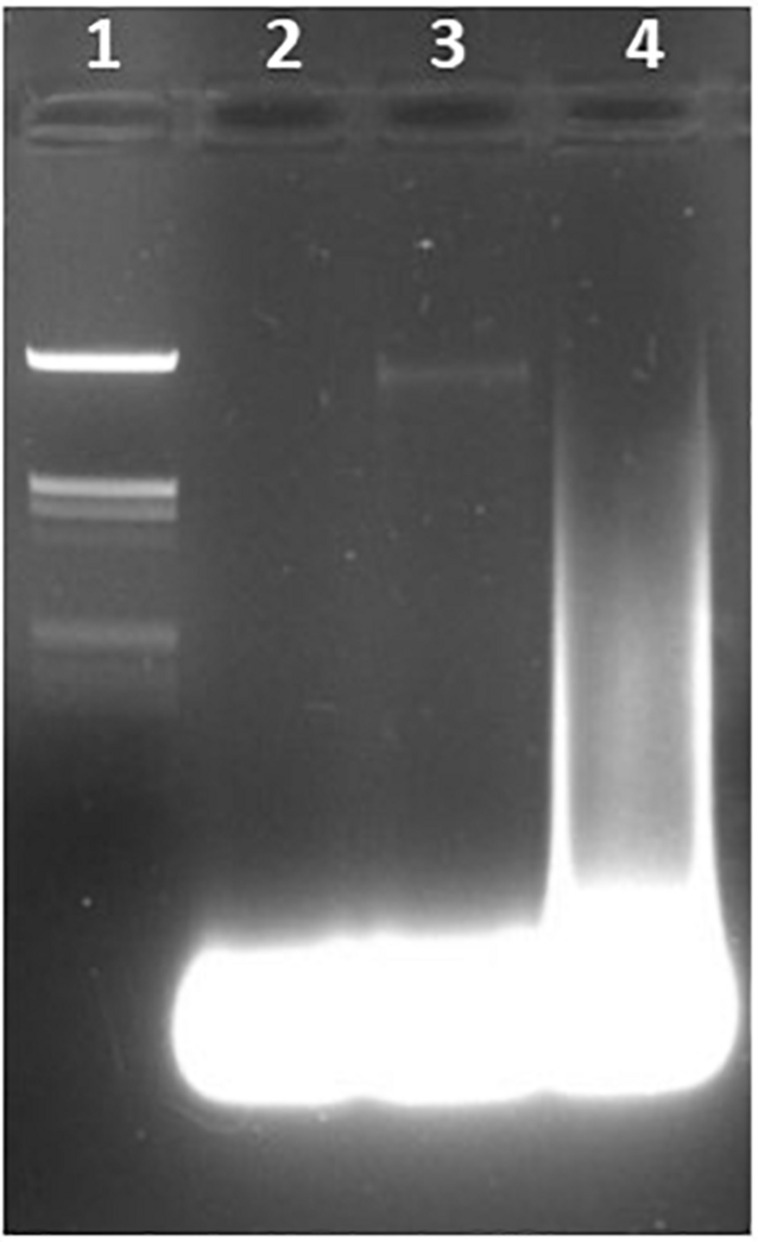
Detection of the 23S rRNA gene of *Enterococcus faecalis* using LAMP. Lane 1, lambda *Hin*dIII marker. Lane 2, water (negative control). Lane 3, *E. coli*. Lane 4, *E. faecalis* DNA.

As a first step toward the incorporation of LAMP in an URE, students performed amplifications using column-purified DNA prepared from cultures of *E. coli*, *S. aureus*, *Salmonella* spp., and *Enterococcus* spp. As shown in Figures 1–4, successful amplification was obtained with all four strains. The *E. coli* and *S. aureus* amplifications were used as a basis for optimization studies, and students were able to demonstrate a range of product yields by temperature variation, with optimal results within the 60–66°C range as expected (Figures 1, 2). LAMP affords multiple additional parameters for student design of optimization studies, including inner/outer primer ratio, magnesium concentration and incubation time, which will be incorporated in future development of the URE.

#### Specificity

Students were able to confirm the specificity of LAMP for *E. coli* (Figure 1), *Salmonella* spp. (Figure 3), and *Enterococcus* (Figure 4), as well as the conservation of the *fimY* target in multiple *Salmonella* strains (Figure 3). Students were also able to observe non-specific elements, such as primer bands and chromosomal DNA (Figure 1B, lane 2). An ambiguous result was obtained with *Streptococcus* (*Enterococcus*) (Figure 3A, lane 5), likely reflecting contamination and/or excess chromosomal DNA. The exquisite sensitivity of LAMP enhances the opportunity for students to observe such false positives, providing opportunity for critical thinking and troubleshooting.

#### DNA-Preparation Method

To assess the dispensability of column-based DNA preparation in the pedagogical use of LAMP, students were given a blind series of sixteen distinct *S. aureus* strains, of which six were MRSA, and used both column and boiling methods for each strain, prior to performing *mecA* LAMP to identify MRSA strains. Similar performance was observed with the two DNA preparation methods: in each case, two MRSA strains were correctly identified (one of which was identified by both methods) and in each case there were four false negatives (data not shown). Overall, these data are supportive of the utilization of LAMP in classroom-based and distance learning settings.

### Amplification From Environmental Samples

**FIGURE 5:**
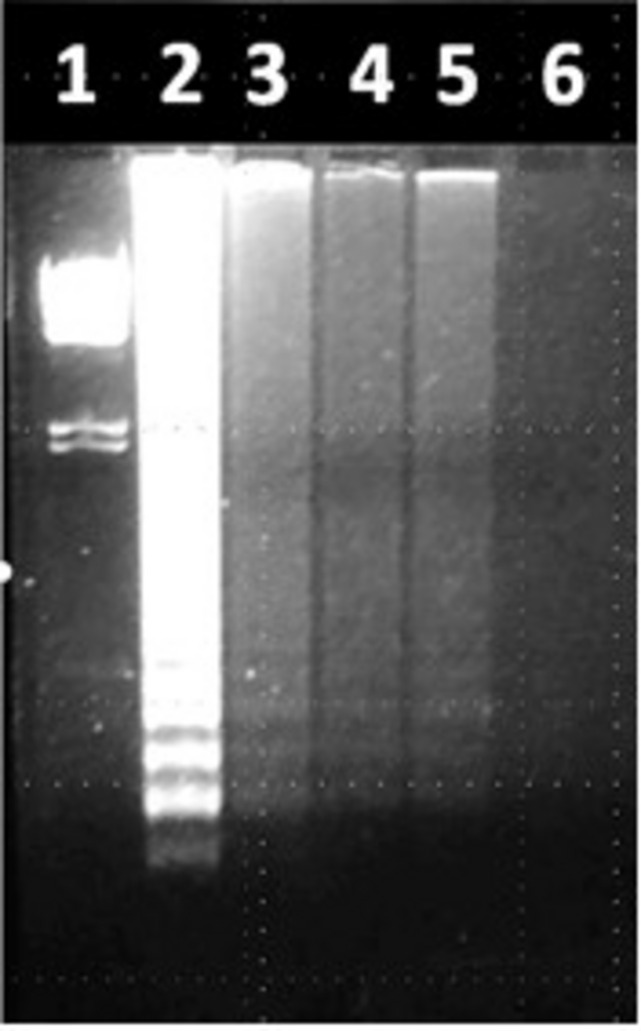
Use of LAMP to detect *Salmonella* spp. in environmental samples (*fimY* target). Lane 1, lambda *Hin*dIII marker. Lane 2, water spiked with *S. typhimurium* (positive control). Lane 3, Alley Pond water. Lane 4, standing water in the QCC parking lot. Lane 5, soil at QCC resuspended in water. Lane 6, distilled water (negative control).

**FIGURE 6:**
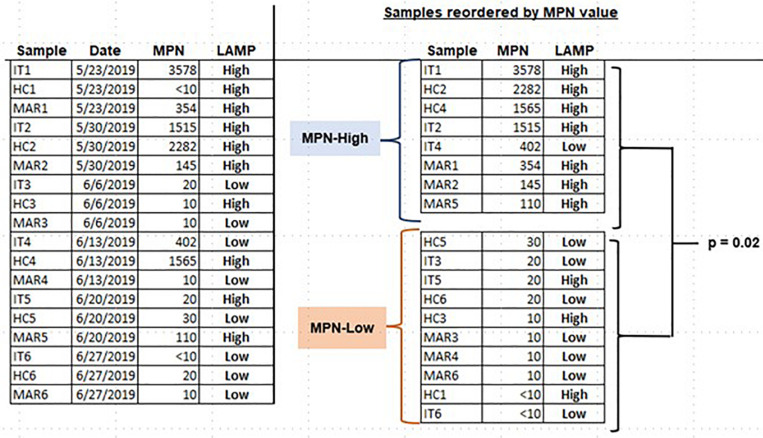
Assessing *Enterococcus* spp. in water samples using LAMP. Water samples were collected at the indicated times from three different locations in the East River in New York City. Samples were tested for *Enterococcus* spp. both by Enterolert (IDEXX) and LAMP. LAMP results were made semi-quantitative by kinetic monitoring of SYBR Green fluorescence (see text). The association of high LAMP values with high MPN values was assessed by one-tailed Fisher’s exact test.

Our students used the LAMP assays they had established to assess environmental samples for either *Salmonella* spp. or *Enterococcus* spp. Enrichments were not performed, since the goal was to determine whether LAMP assay was adequate to allow students to detect these microorganisms with minimal processing. *Salmonella* spp. was detected in a water sample from a kettle pond in an urban park (Alley Pond, Bayside, NY, United States), as well as in standing water and soil samples within the college campus (Figure 5). *Enterococcus* was assessed by LAMP in a series of samples taken over several months from three locations in an urban waterway (East River in New York City). *Enterococcus* levels in these samples were also quantitatively assessed by defined substrate technology (Enterolert; IDEXX). To allow the students to more objectively relate the Enterolert data to their qualitative LAMP findings, the LAMP results were made semi-quantitative by determining the reaction time (T_*t*_) required to achieve a threshold level of SYBR Green fluorescence. The T_*t*_ values were used to divide the samples into a LAMP-High group (T_*t*_ < 22) and a LAMP-Low group (T_*t*_ > 30). The samples were also divided on the basis of the Enterolert data into groups that were either high or low for most probable number (MPN, comparable to CFU). The MPN-High group had MPN values of 110–3,578) while the MPN-Low group had MPN <10–30. It was observed that seven of eight MPN-High samples were LAMP-High, compared to three of ten samples in the MPN-Low group. This difference was significant at *p* = 0.02 by Fisher’s exact text (Figure 6). These results show that students are able to apply LAMP semi-quantitatively to assess environmental levels of bacterial pathogens as part of an URE.

## Discussion

We sought a new approach to developing UR projects to identify common microorganisms that can be detected in environmental samples. Standard methods for the detection of these microorganisms involve culturing and isolating the bacteria, which is a time-consuming and laborious process. Amplification of genomic DNA by PCR is considerably faster but requires an expensive thermocycler and somewhat expensive reagents. We propose LAMP as a promising alternative method to detect microorganisms, as it is rapid, inexpensive with respect to both equipment and reagents, and does not require much effort to set up (Tables 2, 3 and Supplementary Figure S1).

**TABLE 2:** Materials required to set up LAMP and PCR.

	**Power supply ($600)**	**Electrophoresis tank ($400)**	**UV transilluminator ($1,000–5,000)**	**Thermocycler ($3,000)**	**Water bath ($500)**	**Total cost**
PCR	✓	✓	✓	✓	—	$5,500
LAMP	✓	✓	✓	—	✓	$2,500
Simple LAMP*	—	—	—	—	✓	$500
At-home LAMP*	—	—	—	—	—	$0

*LAMP using colorimetric readout and samples prepared by boiling.

**TABLE 3:** Reagent cost comparison between LAMP, PCR, and qPCR.

	**Real time PCR (per reaction)**	**Traditional PCR (per reaction)**	**LAMP (per reaction)**
Enzyme (*Taq* or *Bst*) SYBR^TM^ Green PCR Master mix 1 ml = $125 (ThermoFisher) Taq Polymerase 500 Unit (U) = $236 0.25–2.5 U/50 μl reaction (ThermoFisher) *Bst* Enzyme 8,000 U/ml = $70 (New England Biolabs)	$2.50	– $0.47 per Unit	– – $0.07
Plates or Strip 300 strips = $126	$0.42/strip $0.0525/well	–	–
Microfuge tubes (ThermoFisher) 500 = $22	$0.044	$0.044	$0.044
PCR tubes 1,000 = $119 (ThermoFisher)	–	$0.12	–
Primers: (0.1–1 μM) 100 μM–$40	Forward and Backward $0.04	Forward and Backward $0.04	F3/B3 = $0.04 LF/FB = $0.04 FIP/BIP = $0.08
dNTP’s 100 mM 4 × 250 μl = $320 (ThermoFisher)		50 μM each 2,000 reactions $0.16	50 μM each 2,000 reactions $0.16
Agarose gel (ThermoFisher) 100 g = $145	1% gel–$1.45 $0.08 per lane	1% gel–$1.45 $0.08 per lane	1% gel–$1.45 $0.08 per lane
TOTAL*	$2.71^¥^	$0.91^8^	$0.52^8^

*This is an underestimate as plastic tips/DNA markers/buffers are not calculated. ^¥^Most real time PCR reactions are run in triplicate with appropriate internal controls. ^8^Samples can be run in duplicate. The estimate here is within reported data for the cost per LAMP reaction to be approximately 60–70 cents (1) while most DNA amplification cost between $1–2 per reaction and $5–7 per reaction for real time PCR (2).

We demonstrate here that, even in the undergraduate setting, the LAMP technique can be effectively used to replace PCR to help identify bacterial microorganisms. Our data demonstrate that students with modest training in molecular biology can be involved in authentic research aimed at detecting microbes in unknown environmental samples. We have trained eleven students over the course of three semesters to successfully use this technique to detect environmental microbes. We used LAMP to amplify four common and clinically relevant environmental bacteria: *E. coli*, *S. enteritidis*, *Enterococcus*, and *S. aureus*. All of the data presented here were generated in student-performed studies conducted within a semester time frame, as part of an independent study research course. The students not only acquired basic laboratory proficiency but were able to engage the primary literature.

**FIGURE 7:**
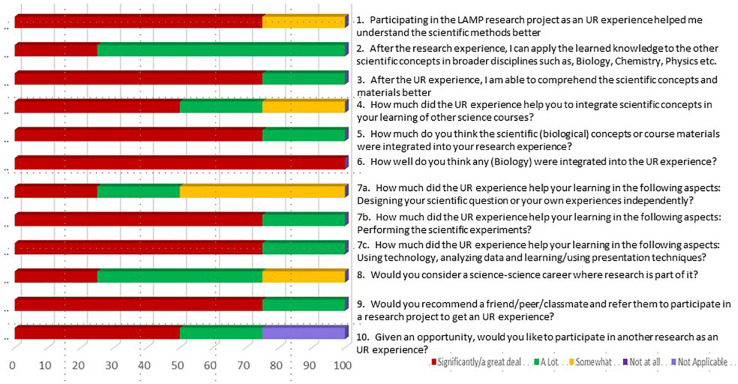
Survey of student attitudes. An anonymous survey was conducted using Google forms.

Two of our student researchers went on to present their findings at regional and national conferences and won prizes for their posters. One student went on to obtain a Research Experiences for Undergraduates (REU) internship in a prestigious 4-year college. Our preliminary data illustrate the abundant opportunities provided by LAMP technology for students to develop skills in critical thinking and experimental design. Students can optimize multiple parameters, troubleshoot unexpected or discrepant findings, develop comparisons with alternative assays, and test hypotheses with respect to the incidence and distribution of multiple microbial microorganisms. A student response survey data shows that majority of the students had a positive and engaging UR experience (Figure 7).

Loop-mediated isothermal amplification technology shares with PCR a notable potential to connect URE with important public health issues. Since *E. coli*, *Salmonella* spp., *Enterococcus* spp., and *S. aureus* spread via contaminated food or water, as well as via infected individuals, environmental detection of these microorganisms can limit the occurrence and magnitude of outbreaks (Curran, 2017). Notably, LAMP has emerged as a relevant technology for the detection of norovirus, *Clostridium perfringens*, and *Campylobacter jejuni*, three of the five most prevalent foodborne pathogens in the United States (Fukuda et al., 2006; Yamazaki et al., 2009; Kaneko et al., 2011), and has recently been used to detect COVID-19 (Kashir and Yaqinuddin, 2020). Students could use LAMP to detect these and other types of microorganisms in the course of developing their own projects. The only additional reagents required would be new primers.

Incorporating authentic research in undergraduate curriculum is challenging due to technical demands and time constraints (Smyth, 2017). Implementing LAMP-based research projects as a CURE is a potential solution. Our data show that both cultures and environmental samples can be effectively processed for LAMP by simple boiling, which should greatly facilitate the design of these experiences in a classroom setting. We intend to implement LAMP-based CUREs in several major and non-major Biology courses using environmental samples. Students will be encouraged to formulate hypotheses as to which environmental sites or site categories are likely to harbor the greatest prevalence of pathogenic microorganisms. Faculty mentors will assist by engaging students in learning about microbes in the context of urban ecology and public health. Pre/post surveys will be used to assess impact on student understanding of scientific method and microbial biology, as well as attitudes toward potential STEM careers.

**FIGURE 8:**
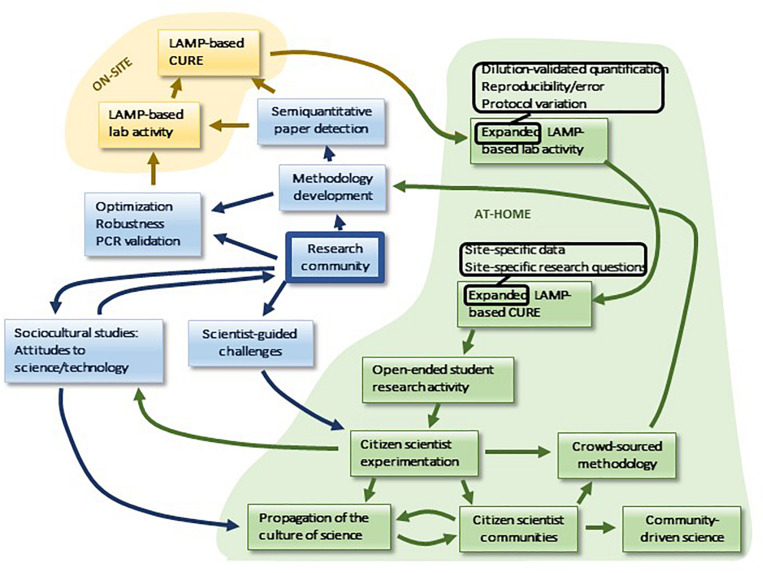
A potential usage of LAMP in distance learning.

Finally, a unique feature of LAMP is the potential for its use in distance learning, and more broadly for both student- and community-based research activity, presenting an unusual opportunity for the expansion of public engagement in science as well as interaction with the research community. This potential developmental sequence is summarized in Figure 8. In the context of distance learning, LAMP is uniquely suited to at-home use in a manner that parallels on-site procedures. All components are stable, including the *Bst* polymerase, which displays remarkable stability (Meridian Bioscience). Any household with an oven, a pot and a thermometer can establish the necessary constant-temperature incubation conditions, and colorimetric readout is readily accomplished. The boiling method for DNA prep can be readily adapted for at-home implementation: inexpensive oven-safe evaporating dishes can be used for sample concentration, substituting for centrifugation. Non-specific products are expected to be rare due to the requirement for hybridization at six independent sequences in the target. Furthermore, it is likely that paper strip-based readout methods will become available that will facilitate semiquantitative analysis (Hongwarittorrn et al., 2017). Quantitation by such methods, as well as by dilution, will allow at-home students to pursue expanded LAMP-based lab activities, including error analysis and protocol variations, which take advantage of the time and flexibility afforded by flipped lab designs. Similarly, at-home CUREs offer expanded possibilities in comparison to on-site research, as students can develop research questions and collect samples in a manner that is tailored to the unique as well as shared aspects of their individual locations. Continual access to the home “laboratory” is likely to encourage students to generate additional research projects in an open-ended manner, providing an unusual opportunity for training in project design at the undergraduate level.

Moreover, the possibilities for at-home LAMP are not limited to the student body, which may serve as a vanguard for the encouragement of similar scientific research activity in communities. Therein potentially lies a unique opportunity for the development of the public culture of science. For example, while it is becoming increasingly common for scientists to crowdsource sample acquisition from the lay public, LAMP is perhaps the unique technology that may permit lay crowdsourcing, not only of samples, but also of methodological data. Lay LAMP-tinkerers may make a contribution to the professional research community, if their numbers outweigh their lack of scientific sophistication. Scientists may contribute to and help guide this process by offering research challenges to such lay communities. Communities of citizen scientists can potentially organize projects of their own, and they may also be a source of sociocultural data for the professional study of the culture of science (Figure 8). By engaging the public this way, we anticipate that this may increase understanding and engagement with science as well as broadening access to and increasing inclusiveness in the process of science.

In summary, incorporation of LAMP in URE has many benefits, including low cost, speed, ease of training, and the ability to engage undergraduates in meaningful UREs. Moreover, beyond these technical advantages, LAMP has unusual promise as a technology for expanding student and community experience in authentic scientific inquiry. UR has become a hallmark national trend as a component of HIP pedagogy. We have been able to observe the value of UR to undergraduate education on a personal level, noting the growth in self-confidence, independence and communication skills in our students as they progress through the program. Further development of LAMP-based approaches to UR will allow students to gain first-hand knowledge of STEM careers, and more broadly to apply newly acquired skills and experience to their further education and professional development.

## Data Availability Statement

The original contributions presented in the study are included in the article/Supplementary Material, further inquiries can be directed to the corresponding author/s.

## Author Contributions

All authors contributed to the design of the experiments and agreed to working on different projects. AN planned the project, supervised, and wrote an initial draft. AO revised and rewrote the manuscript, analyzed data, and added a figure. DS and MT contributed to the revision. AN, AO, and MT designed and implemented IRB-approved student survey. CP and MK provided discussion and strategic approach to LAMP research regarding *Enterococcus* and *Salmonella*, respectively. All the students (KP, GL, DM, OB, MF, JS, WK, EC, YK, TN, MF, JE, and MM) performed experiments, data analysis and participated in manuscript preparation. All authors critically discussed and contributed to the final version.

## Conflict of Interest

The authors declare that the research was conducted in the absence of any commercial or financial relationships that could be construed as a potential conflict of interest.

## Supplementary Material

Supplementary Figure 1 Comparison of DNA amplification using PCR **(A)** or LAMP **(B)**. PCR requires the use of a thermocycler and electrophoresis equipment **(A)**. In LAMP, self-priming obviates the need for a thermocycler, and colorimetric readout provides an alternative to electrophoresis **(B)**.

Supplementary Figure 2 Schematic diagram of LAMP. *Part 1.* Template DNAwith primer target sequences indicated (F3c, F2c, and F1c). *Part 2.* Primer FIPbinds to target sequence F2c, with a 5′ overhang containing F1c (top panel). Primer F3 is used to unzip and release strand A2 (middle panel), which then formsa loop at its 5′ end via F1c-F1 complementarity (bottom panel). *Part 3.* The 3′ endof strand A2 is targeted by primer BIP, followed by unzipping with primer B3 torelease strand A4, which can forms loops at both 5′ and 3′ ends. *Part 4.* Theresulting dumbbell (strand A4) is comparable to a similar dumbbell (strand B4)formed by a corresponding series of events (not shown) beginning with targetingof B2c in strand B (*Part 1*). *Part 5.* Loop amplification yields concatemers of targetsequences, beginning either with strand A4 (*Part 5a*) or with strand B4 (*Part 5b*).

## References

[B1] Azinheiro S. Carvalho J. Prado M. Garrido-Maestu A. (2018). Evaluation of different genetic targets for *Salmonella enterica* Serovar Enteritidis and Typhimurium, using loop-mediated isothermal amplification for detection in food samples. *Front. Sustain Food Syst.* 2:5 10.3389/fsufs.2018.00005

[B2] Chen C. Zhao Q. Guo J. Li Y. Chen Q. (2017). Identification of Methicillin-Resistant Staphylococcus aureus (MRSA) Using Simultaneous Detection of mecA, nuc, and femB by Loop-Mediated Isothermal Amplification (LAMP). *Curr. Microbiol.* 74 965–971. 10.1007/s00284-017-1274-2 28573341

[B3] Curran E. (2017). Infection outbreaks in care homes: prevention and management. *Nurs. Times* 113 18–21.

[B4] De Medici D. Croci L. Delibato E. Pasquale S. Filetici E. Laura T. (2003). Evaluation of DNA extraction methods for use in combination with SYBR Green I real-time PCR to detect *Salmonella enterica* Serotype Enteritidis in poultry. *Appl. Environ. Microbiol.* 69 3456–3461. 10.1128/aem.69.6.3456-3461.2003 12788750 PMC161507

[B5] Fukuda S. Takao S. Kuwayama M. Shimazu Y. Miyazaki K. (2006). Rapid detection of norovirus from fecal specimens by real-time reverse transcription-loop-mediated isothermal amplification assay. *J. Clin. Microbiol.* 44 1376–1381. 10.1128/jcm.44.4.1376-1381.2006 16597865 PMC1448634

[B6] Hill J. Beriwal S. Chandra I. Paul V. K. Kapil A. Singh T. (2008). Loop-mediated isothermal amplification assay for rapid detection of common strains of *Escherichia coli*. *J. Clin. Microbiol.* 46 2800–2804. 10.1128/jcm.00152-08 18550738 PMC2519505

[B7] Hongwarittorrn I. Chaichanawongsaroj N. Laiwattanapaisal W. (2017). Semi-quantitative visual detection of loop mediated isothermal amplification (LAMP)-generated DNA by distance-based measurement on a paper device. *Talanta* 175 135–142. 10.1016/j.talanta.2017.07.019 28841970

[B8] Hu L. Ma L. M. Zheng S. He X. Hammack T. S. Brown E. W. (2018). Development of a novel loop-mediated isothermal amplification (LAMP) assay for the detection of *Salmonella* ser. Enteritidis egg products. *Food Control* 88 190–197. 10.1016/j.foodcont.2018.01.006

[B9] Kaneko I. Kawana T. Fukushima E. Suzutani T. (2007). Tolerance of loop-mediated isothermal amplification to a culture medium and biological substances. *J. Biochem. Biophys. Methods* 70, 499–501. 10.1016/j.jbbm.2006.08.008 17011631

[B10] Kaneko I. Miyamoto K. Mimura K. Yumine N. Utsunomiya H. Akimoto S. (2011). Detection of enterotoxigenic Clostridium perfringens in meat samples by using molecular methods. *Appl. Environ. Microbiol.* 77 7526–7532. 10.1128/aem.06216-11 21890671 PMC3209162

[B11] Kashir J. Yaqinuddin A. (2020). Loop mediated isothermal amplification (LAMP) assays as a rapid diagnostic for COVID-19. *Med. Hypotheses* 141:109786. 10.1016/j.mehy.2020.109786 32361529 PMC7182526

[B12] Kuh G. D. (2008). *High-Impact Educational Practices: What They Are, Who Has Access to Them, and Why They Matter.* Washington, D.C: Association of American Colleges and Universities.

[B13] Kuh G. D. O’Donnell K. (2013). *Ensuring Quality & Taking High-Impact Practices to Scale with Case Studies by Sally Reed.* Washington, DC: Association of American Colleges and Universities.

[B14] Law J. W. Ab Mutalib N. S. Chan K. G. Lee L. H. (2014). Rapid methods for the detection of foodborne bacterial pathogens: principles, applications, advantages and limitations. *Front. Microbiol.* 5:770. 10.3389/fmicb.2014.00770 25628612 PMC4290631

[B15] Lin Q. Xu P. Li J. Chen Y. Feng J. (2017). Direct bacterial loop-mediated isothermal amplification detection on the pathogenic features of the nosocomial pathogen - Methicillin resistant Staphylococcus aureus strains with respiratory origins. *Microb. Pathog.* 109 183–188. 10.1016/j.micpath.2017.05.044 28578093

[B16] Martzy R. Kolm C. Brunner K. Mach R. L. Krska R. Šinkovec H. (2017). A loop-mediated isothermal amplification (LAMP) assay for the rapid detection of Enterococcus spp. in water. *Water Res.* 122 62–69. 10.1016/j.watres.2017.05.023 28591662

[B17] Mori Y. Notomi T. (2009). Loop-mediated isothermal amplifi cation (LAMP): a rapid, accurate, and cost-effective diagnostic method for infectious diseases. *J. Infect. Chemother.* 15 62–69. 10.1007/s10156-009-0669-9 19396514 PMC7087713

[B18] Smyth D.S. (2017). An Authentic Course-Based Research Experience in Antibiotic Resistance and Microbial Genomics. *Sci. Educ. Civic Engage.* 9:59.

[B19] Tang T. Cheng A. Wang M. Li X. He Q. Jia R. (2012). Development and clinical verification of a loop-mediated isothermal amplification method for detection of *Salmonella* species in suspect infected ducks. *Poult. Sci.* 91, 979–986. 10.3382/ps.2011-01992 22399738

[B20] Wang X. R. Wu L. F. Wang Y. Ma Y. Y. Chen F. H. Ou H. L. (2015). Rapid detection of Staphylococcus aureus by loop-mediated isothermal amplification. *Appl. Biochem. Biotechnol.* 175 882–891. 10.1007/s12010-014-1328-x 25349088

[B21] Warghane A. Misra P. Bhose S. Biswas K. K. Sharma A. K. Reddy M. K. (2017). Development of a simple and rapid reverse transcription-loop mediated isothermal amplification (RT-LAMP) assay for sensitive detection of *Citrus tristeza virus*. *J. Virol. Methods* 250 6–10. 10.1016/j.jviromet.2017.09.018 28941614

[B22] Yamazaki W. Taguchi M. Kawai T. Kawatsu K. Sakata J. Inoue K. (2009). Comparison of loop-mediated isothermal amplification assay and conventional culture methods for detection of Campylobacter jejuni and Campylobacter coli in naturally contaminated chicken meat samples. *Appl. Environ. Microbiol.* 75 1597–1603. 10.1128/aem.02004-08 19139242 PMC2655443

