# Supplementary figures and images for: Loop-Mediated Isothermal Amplification (LAMP) as a Rapid, Affordable and Effective Tool to Involve Students in Undergraduate Research
Source: Front Microbiol. 2020 Dec 9;11:603381. doi: 10.3389/fmicb.2020.603381 (PMC7756096; doi:10.3389/fmicb.2020.603381)

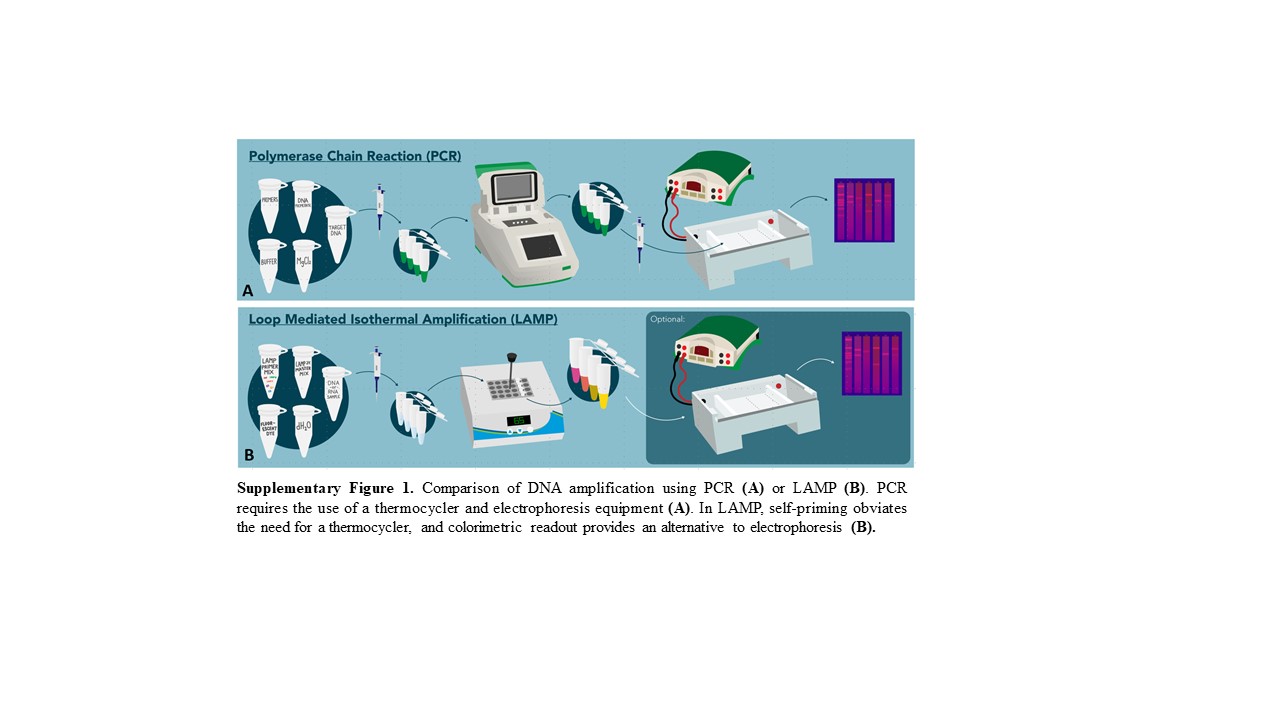

Supplement: Supplementary Figure 1 — Comparison of DNA amplification using PCR (A) or LAMP (B). PCR requires the use of a thermocycler and electrophoresis equipment (A). In LAMP, self-priming obviates the need for a thermocycler, and colorimetric readout provides an alternative to electrophoresis (B). [file Image_1.jpg]
