# Supplementary material for: Loop-Mediated Isothermal Amplification (LAMP) as a Rapid, Affordable and Effective Tool to Involve Students in Undergraduate Research
Source: Front Microbiol. 2020 Dec 9;11:603381. doi: 10.3389/fmicb.2020.603381 (PMC7756096; doi:10.3389/fmicb.2020.603381)

LAMP (Loop-Mediated Isothermal Amplification)

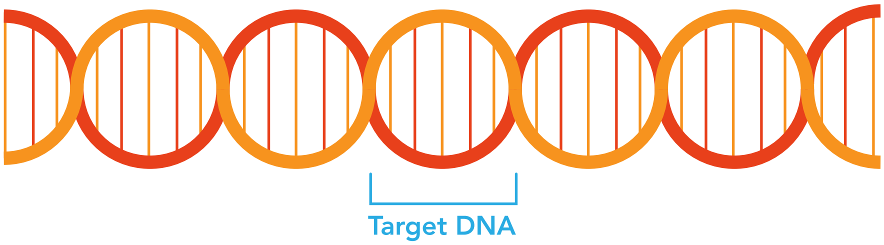

Part 1: Denaturation

Step 1. Denaturing unzips the template DNA

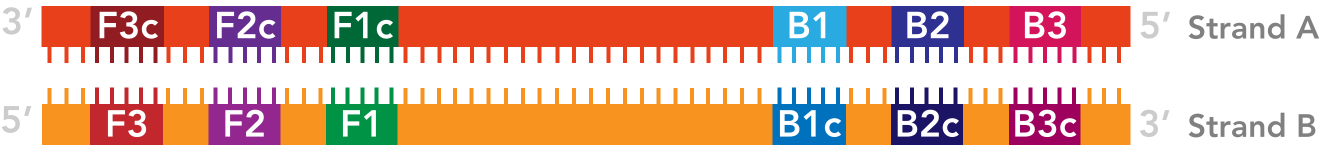

Part 2: Setting up the left side of the dumbell

Foward Inner Primer or FIP (F1c & F2) makes complementary DNA of template strand.

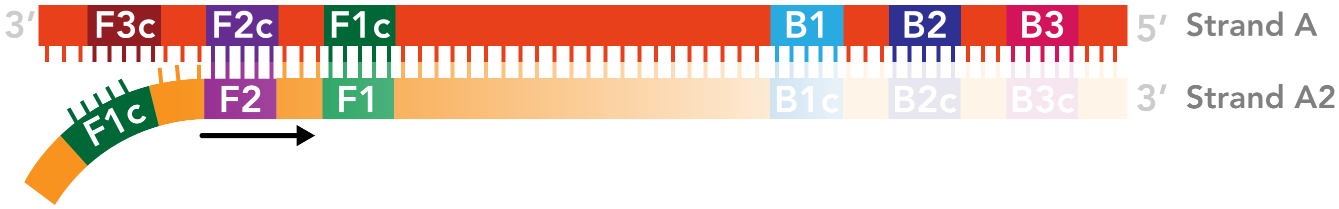

Forward Outer Primer (F3) unzips complementary strand from the template strand.

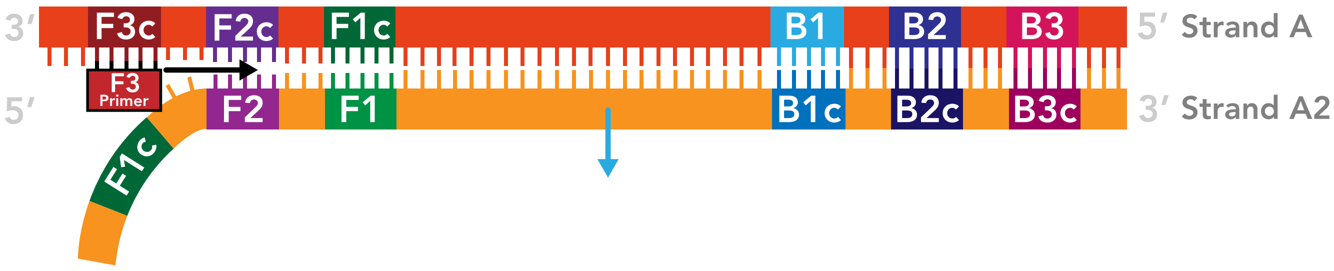

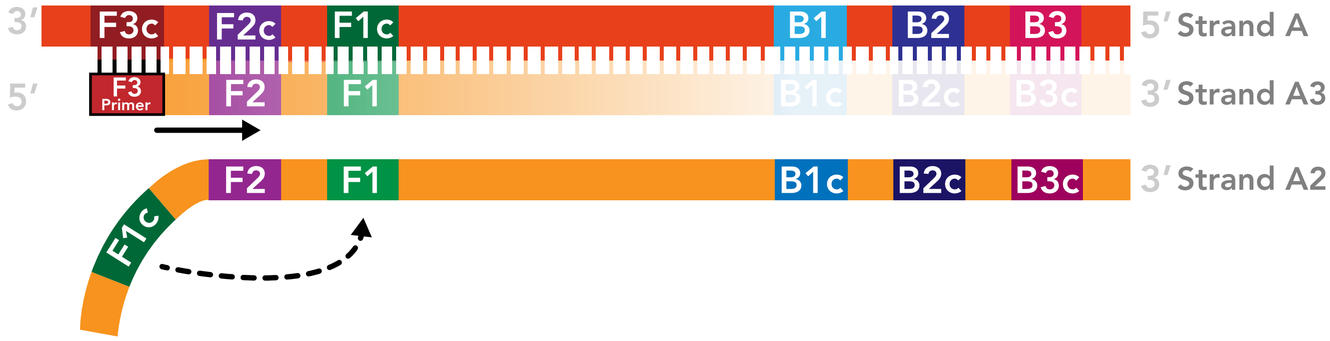

Part 3: Setting up the right side of the dumbell

Backward Inner Primer or BIP (B2 & B1c) makes complementary DNA of new template.

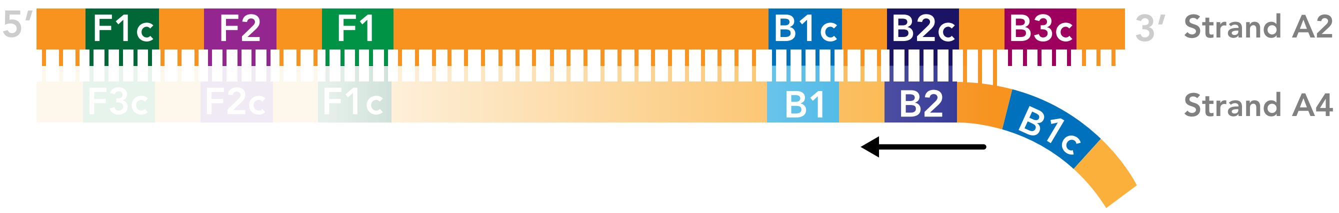

Backward Outer Primer (B3) unzips the new complementary strand.

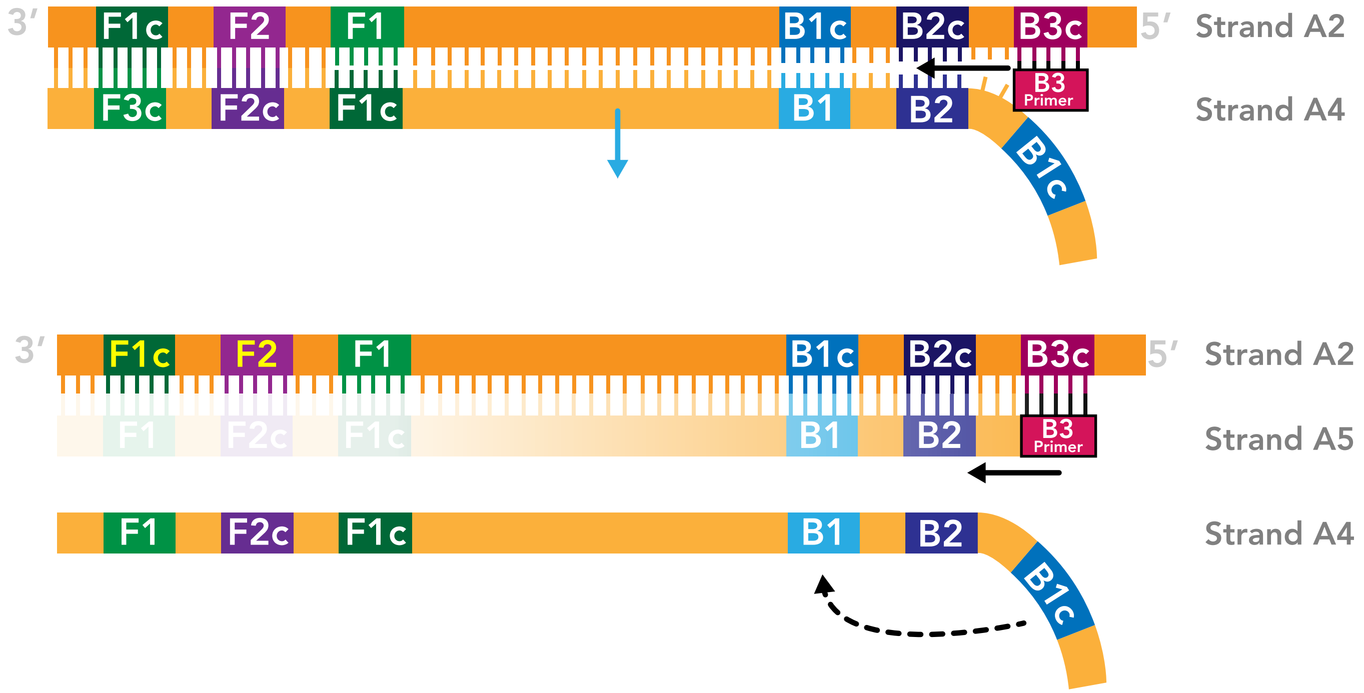

Part 4: Using the dumbell for amplification

F1 & F1c and B1 & B1c complementary regions bind to form "dumbell" shape.

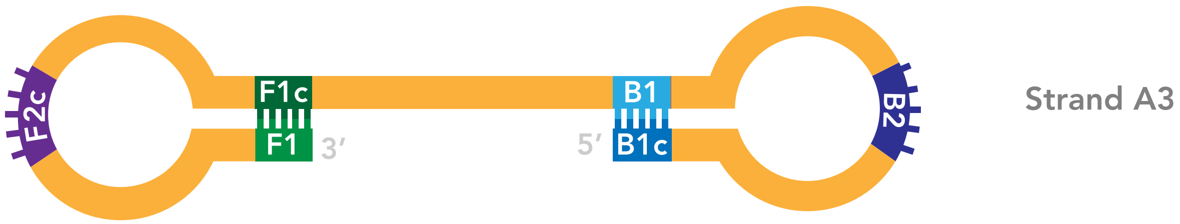

The same process occurs with the other strand of template DNA (Strand B).

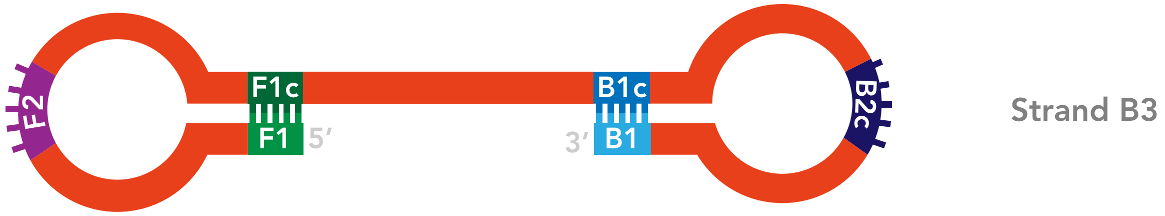

Part 5a: Loop Amplification

The FIP and the BIP bind to the 3′ ends of the dumbell and begin amplification creating concatamers of different lengths with even more binding sites for the FIP and the BIP. Below is a representation of the FIP binding to the dumbell structure and beginning to amplify the DNA.

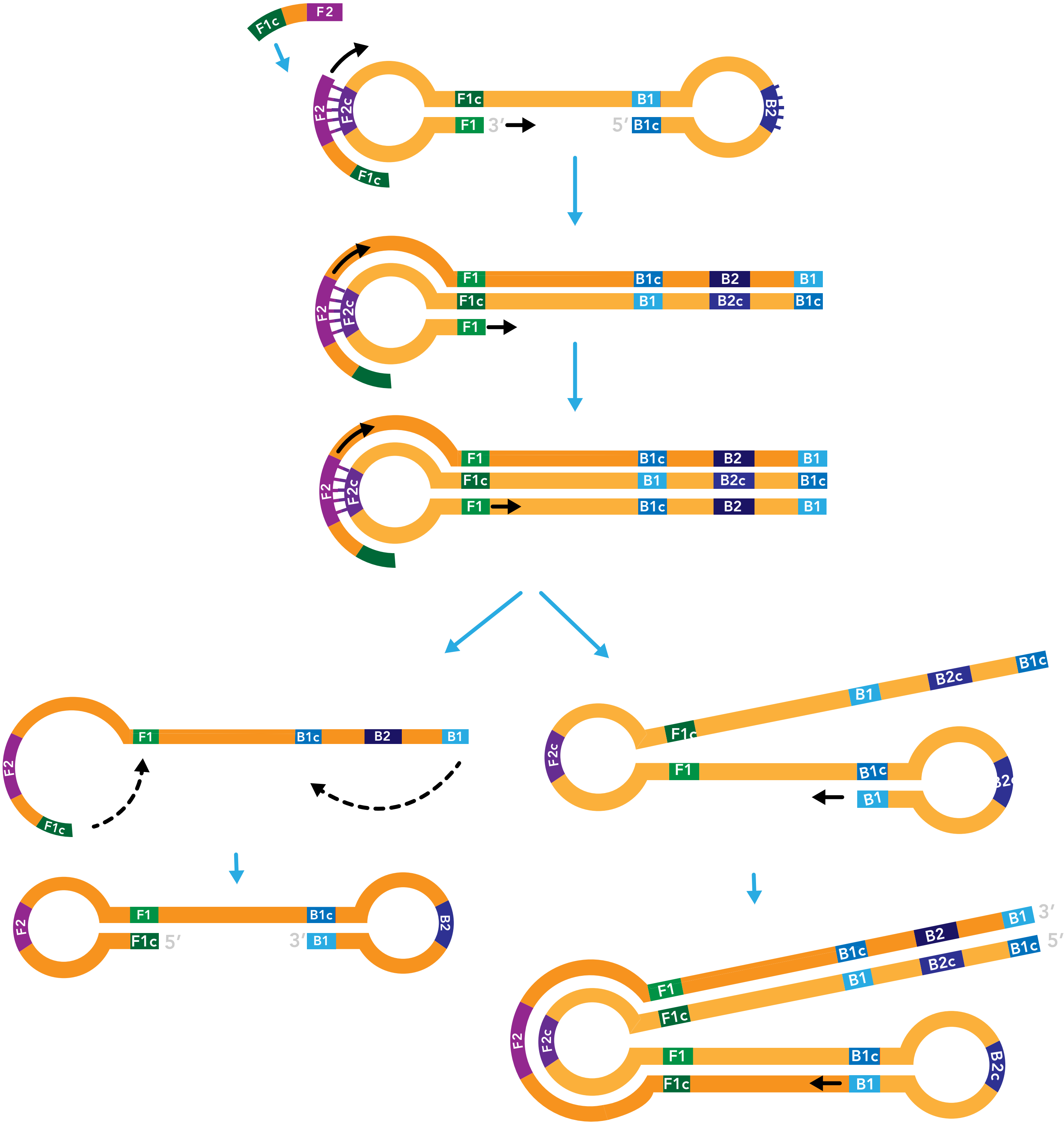

Part 5b: Loop Amplification

The FIP and the BIP bind to the 3′ ends of the dumbell and begin amplification creating concatamers of different lengths with even more binding sites for the FIP and the BIP. Below is a representation of the BIP binding to the dumbell structure and beginning to amplify the DNA.

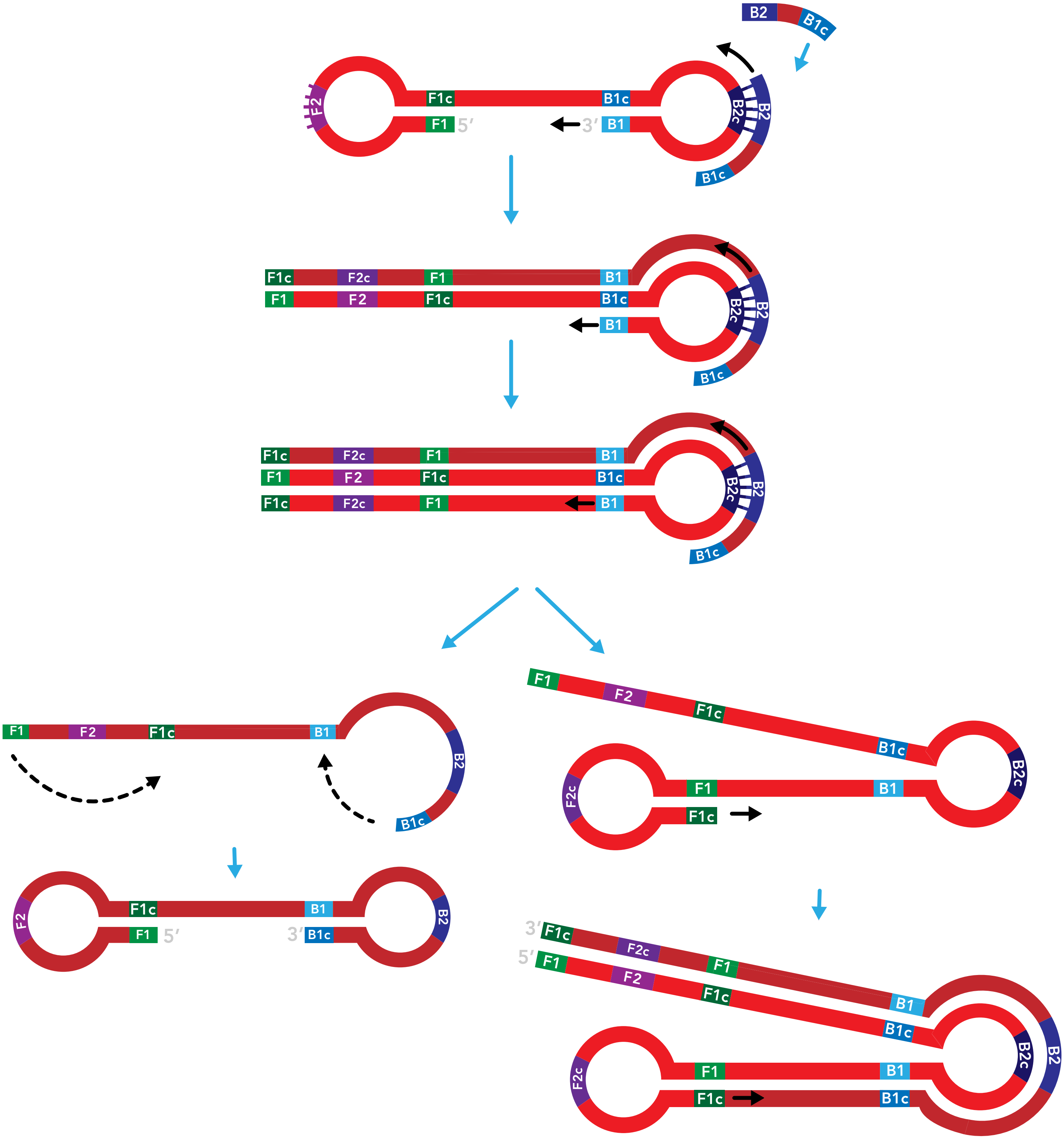

Supplement: Supplementary Figure 2 — Schematic diagram of LAMP. Part 1. Template DNAwith primer target sequences indicated (F3c, F2c, and F1c). Part 2. Primer FIPbinds to target sequence F2c, with a 5′ overhang containing F1c (top panel). Primer F3 is used to unzip and release strand A2 (middle panel), which then formsa loop at its 5′ end via F1c-F1 complementarity (bottom panel). Part 3. The 3′ endof strand A2 is targeted by primer BIP, followed by unzipping with primer B3 torelease strand A4, which can forms loops at both 5′ and 3′ ends. Part 4. Theresulting dumbbell (strand A4) is comparable to a similar dumbbell (strand B4)formed by a corresponding series of events (not shown) beginning with targetingof B2c in strand B (Part 1). Part 5. Loop amplification yields concatemers of targetsequences, beginning either with strand A4 (Part 5a) or with strand B4 (Part 5b). [file Image_2.pdf]
